# Three Main Mutational Pathways in HIV-2 Lead to High-Level Raltegravir and Elvitegravir Resistance: Implications for Emerging HIV-2 Treatment Regimens

**DOI:** 10.1371/journal.pone.0045372

**Published:** 2012-09-18

**Authors:** Robert A. Smith, Dana N. Raugi, Charlotte Pan, Matthew Coyne, Alexandra Hernandez, Brad Church, Kara Parker, James I. Mullins, Papa Salif Sow, Geoffrey S. Gottlieb for the University of Washington–Dakar HIV-2 Study Group

**Affiliations:** 1 Department of Pathology, University of Washington, Seattle, Washington, United States of America; 2 Department of Medicine, Division of Allergy and Infectious Diseases, University of Washington, Seattle, Washington, United States of America; 3 College of Arts and Sciences, University of Washington, Seattle, Washington, United States of America; 4 Department of Microbiology, University of Washington, Seattle, Washington, United States of America; 5 Clinique des Maladies Infectieuses Ibrahima DIOP Mar, Centre Hospitalier Universitaire de Fann, Université Cheikh Anta Diop de Dakar, Dakar, Senegal; Centro de Biología Molecular Severo Ochoa (CSIC-UAM), Spain

## Abstract

Human immunodeficiency virus type 2 (HIV-2) is intrinsically resistant to non-nucleoside reverse transcriptase inhibitors and exhibits reduced susceptibility to several of the protease inhibitors used for antiretroviral therapy of HIV-1. Thus, there is a pressing need to identify new classes of antiretroviral agents that are active against HIV-2. Although recent data suggest that the integrase strand transfer inhibitors raltegravir and elvitegravir may be beneficial, mutations that are known to confer resistance to these drugs in HIV-1 have been reported in HIV-2 sequences from patients receiving raltegravir-containing regimens. To examine the phenotypic effects of mutations that emerge during raltegravir treatment, we constructed a panel of HIV-2 integrase variants using site-directed mutagenesis and measured the susceptibilities of the mutant strains to raltegravir and elvitegravir in culture. The effects of single and multiple amino acid changes on HIV-2 replication capacity were also evaluated. Our results demonstrate that secondary replacements in the integrase protein play key roles in the development of integrase inhibitor resistance in HIV-2. Collectively, our data define three major mutational pathways to high-level raltegravir and elvitegravir resistance: *i*) E92Q+Y143C or T97A+Y143C, *ii*) G140S+Q148R, and *iii*) E92Q+N155H. These findings preclude the sequential use of raltegravir and elvitegravir (or vice versa) for HIV-2 treatment and provide important information for clinical monitoring of integrase inhibitor resistance in HIV-2–infected individuals.

## Introduction

Human immunodeficiency virus type 2 (HIV-2) is believed to have originated in West Africa, where the virus is endemic, and has spread to other areas with socio-economic ties to the region [1]. Although HIV-2 infection typically involves a prolonged asymptomatic phase, significant numbers of HIV-2 patients eventually progress to AIDS and may benefit from antiretroviral (ARV) therapy [2], [3]. Unfortunately, treatment of HIV-2 is complicated by a number of factors including the intrinsic resistance of the virus to non-nucleoside reverse transcriptase inhibitors, the diminished sensitivity of HIV-2 to most of the protease inhibitors used against HIV-1, and the rapid emergence of broad-class nucleoside analog resistance [4], [5], [6], [7], [8], [9]. In addition, newer ARV drugs that exhibit potent and durable anti–HIV-1 activity (*i.e.*, tenofovir disoproxil fumarate/emtricitabine and ritonavir-boosted lopinavir) have only recently become available in West Africa. The use of suboptimal ARVs for HIV-2 treatment has resulted in a high frequency of multiclass drug resistance in the region [4]. Non-protease inhibitor-based first-line options are needed for HIV-2, in addition to new second-line ARVs, because resistance and adverse effects have left many HIV-2–infected individuals with few (if any) options for effective treatment.

Recent studies suggest that integrase strand transfer inhibitors (INSTIs) could help fill the urgent need for new classes of HIV-2–active ARVs. Raltegravir, elvitegravir and dolutegravir are active against HIV-2 with 50% effective concentrations (EC_50_ values) in the low-nanomolar range [10], [11], [12], and limited data suggest that raltegravir-based regimens can initially suppress plasma viral loads in HIV-2–infected individuals [13]. However, as is the case for HIV-1, mutations encoding specific amino acid changes in the HIV-2 integrase protein appear to compromise the durability of raltegravir treatment [13], [14], [15], [16], [17], [18] (Table 1). These outcomes underscore the need to thoroughly characterize the genetic pathways to integrase inhibitor resistance in HIV-2 and to determine the extent of cross-resistance between different inhibitors in the INSTI class.

**Table 1:** Amino acid changes observed in integrase sequences from raltegravir-treated HIV-2 patients.

Author, year, reference	Number of patients studied^a^	Primary INSTI-associated changes observed^b^	Additional changes reported
Garrett et al., 2008, [14]	1	N155H	none
Roquebert et al., 2008, [15]	1	Q148K, Q148R	none
Salgado et al., 2009, [16]	1	N155H	A33A/G, H51H/R, V72I, I84V, A153G, N160K, S163S/G
Xu et al., 2009, [18]	1	N155H, E92Q+N155H, T97A+Y143C^c^	E92G, Q91R, S147G, A153G, H157R, M183I
Charpentier et al., 2011, [13]	7	T97A+Y143C^d^, Q148K^e^, Q148R, G140S+Q148R, E92E/Q+Y143H/R+N155H^e^, T97A+N155H	none
Treviño et. al, 2011, [17]	3	N155H, E92Q, T97A^f^	A153G, S163G/D

a Patients that did not receive raltegravir-containing treatment are not included in this table.

b Genotypic analyses were performed on consensus (bulk) sequences unless otherwise indicated.

c These changes appeared in cloned PCR products amplified from a single patient.

d This combination of replacements was observed in two study subjects.

e Replacements listed for Charpentier et al. are the changes observed at time of virologic failure. Upon further raltegravir treatment, the predominant genotypes in the two patients harboring Q148K/R changed to G140S+Q148R, and the consensus sequence of the patient with E92E/Q+Y143H/R+N155H changed to E92E/A/P/Q+N155H.

f N155H variants emerged in two study subjects. The exact combinations of changes were not specified.

We previously showed that the single amino acid changes Q148R and N155H in HIV-2 integrase confer moderate resistance to raltegravir, whereas the Y143C change had a minimal effect on raltegravir sensitivity [11]. Our current work demonstrates that secondary amino acid changes in the HIV-2 integrase protein play critical roles in the development of raltegravir and elvitegravir resistance. These additional changes cooperate with Y143C, Q148R and N155H to produce more substantial declines in drug susceptibility. Collectively, our findings identify three major mutational pathways to high-level raltegravir and elvitegravir resistance in HIV-2.

## Materials and Methods

Raltegravir was obtained from the National Institutes of Health AIDS Research and Reference Reagent Program. Master stocks of raltegravir (5 mM) were prepared by dissolving 2 mg of drug in 830 µl of sterile distilled water. Serial dilutions of the drug were prepared in sterile distilled water at concentrations ten-fold greater than the final assay concentrations and were stored in 1-ml aliquots at −80°C.

Elvitegravir was provided by Gilead Sciences, Inc (Foster City, California). Concentrations of elvitegravir ≥100 µM were insoluble in water. Therefore, master stocks of the drug (10 mM) were prepared by dissolving 20 mg of elvitegravir powder in 4.47 ml high performance liquid chromatography-grade dimethyl sulfoxide (HPLC-grade DMSO) packaged under argon gas (Alfa Aesar Co., Ward Hill, Massachusetts). Serial ten-fold dilutions of elvitegravir ranging from 0.0001 nM to 10 µM, as well as 40 µM stocks of the drug, were prepared in aqueous solutions containing 10% (vol/vol) HPLC-grade DMSO, yielding working stocks with drug concentrations 10-fold greater than the final elvitegravir concentrations used in the assay. All master stocks and working solutions of elvitegravir were stored in 1-ml aliquots at −80°C.

All integrase variants used in this study were constructed in the full-length pROD9 molecular clone (HIV-2 group A; kindly provided by Michael Emerman, Fred Hutchinson Cancer Research Center, Seattle, Washington). Plasmids encoding amino changes Y143C, Q148R and N155H were generated as previously described [11]. The remaining integrase variants were constructed via site-directed mutagenesis of full-length pROD9 DNA using the QuikChange XL Kit (Stratagene, La Jolla, California). Nucleotide sequences of the mutagenic primers are available upon request. All full-length plasmids were purified using HiSpeed Plasmid Maxi Kits with buffers and reagents from an EndoFree Plasmid Maxi Kit (Qiagen Inc., Valencia, California) and were sequenced across the entire HIV-2–encoding region to ensure that no unintended nucleotide changes were introduced during the mutagenesis procedure.

Virus stocks were prepared via transient transfection of 293T-17 cells using a chloroquine-mediated calcium phosphate co-precipitation method [5]. Measurements of replication capacity were performed using our previously-published MAGIC-5A indicator cell assay [5]. Raltegravir and elvitegravir susceptibilities were quantified in MAGIC-5A cells as previously described [5], [11] or via a simplified procedure that required fewer liquid handling steps. Both protocols yielded comparable EC_50_ values for the inhibition of wild-type HIV-2 ROD9 by raltegravir and elvitegravir (data not shown). For the simplified assay, MAGIC-5A cells were seeded in 48-well plates at 1.5×10^4^ cells per well in 200 µl of complete medium and incubated overnight (30°C, 5% CO_2_). The following morning, the culture fluids were replaced with 200 µl of fresh medium containing 7.5 µg/ml diethylaminoethyl dextran (Sigma-Aldrich Co., St. Louis, Missouri) and appropriate drug dilutions were added (30 µl/well; solvent-only control wells received 30 µl of water or 10% DMSO for raltegravir and elvitegravir, respectively). Following a one-hour incubation to allow drug uptake, the plates were infected with dilutions of virus stocks (70 µl/well) that typically yielded 300–600 focus-forming units (FFU) in the solvent-only control cultures. The plates were then wrapped in plastic film and returned to the incubator. After 40 hours of growth, the culture monolayers were fixed and stained with 5-bromo-4-chloro-3-indolyl-β-D-galactopyranoside (X-gal) as previously described [5], and β-galactosidase–positive foci were counted by light microscopy. EC_50_ values were calculated from dose-response plots using the sigmoidal regression function of Prism (GraphPad Software Inc., San Diego, California). Log_10_-transformed EC_50_ and titer values were tested for statistically significant differences using the analysis of variance function of Prism (ANOVA) with Tukey’s post-test.

## Results

**Figure 1:**
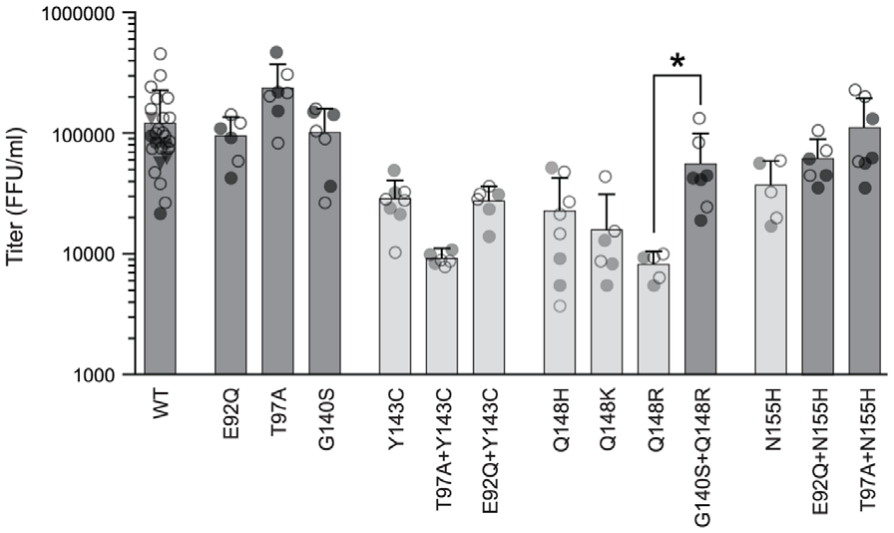
Single-cycle replication capacities of HIV-2 integrase variants. Each datum point is the infectious titer [MAGIC-5A focus-forming units (FFU)/ml] produced by an independent transfection of full-length HIV-2 plasmid DNA into 293T-17 cells. Bars indicate the mean titers for each wild-type (WT) or mutant strain. Light-grey bars indicate variants that are significantly different from WT (*P*>0.05) and * indicates a significant difference between Q148R and G140S+Q148R HIV-2 (*P*>0.01) (ANOVA of log_10_-transformed titers with Tukey’s post-test). Filled and open circles represent the titers produced by two independent plasmid DNA preparations for each genotype; titers from a third preparation of wild-type DNA are shown as inverted triangles. Error bars indicate standard deviations.

To determine the phenotypic consequences of INSTI resistance-associated mutations in HIV-2, we generated base substitutions in the pROD9 infectious molecular clone that correspond to the principal amino acid changes seen in sequences from raltegravir-treated HIV-2 patients (Table 1). We then introduced the mutant plasmids (or wild-type pROD9) into 293T-17 cells via transient transfection and quantified the subsequent release of infectious virus using MAGIC-5A indicator cells (CD4^+^ CCR5^+^ HeLa cells that express β-galactosidase under the control of an HIV-1 LTR promoter). We initially focused our analysis on the effects of single amino acid replacements in the HIV-2 integrase protein. Variants E92Q, T97A, and G140S produced infectious titers that were comparable to wild-type (Figure 1). In contrast, the Y143C, N155H, and Q148H, K, and R changes each conferred statistically-significant declines in infectious virus production, with mean titers 3–15-fold lower than that of wild-type ROD9 (Figure 1). These data demonstrate that treatment-associated mutations at the three key sites involved in raltegravir resistance in HIV-1 (Y143, Q148 and N155; [19]) are deleterious to viral replication in HIV-2, whereas secondary changes alone do not measurably diminish HIV-2 replication capacity.

Next, we constructed infectious clones containing specific combinations of replacements that emerge in HIV-2 in response to raltegravir treatment (Table 1). Notably, the combination of G140S+Q148R resulted in improved replication capacity relative to Q148R alone; titers of the double-mutant were only 2-fold lower than wild type, versus a 15-fold reduction for the Q148R variant (Figure 1). Slight improvements in infectivity were also noted for E92Q+N155H and T97A+N155H relative to N155H alone, although these differences were not statistically significant. In contrast, the infectious titers produced by E92Q+Y143C and T97A+Y143C ROD9 were comparable to or less than those of the Y143C clone (Figure 1), indicating that the E92Q and T97A replacements fail to compensate for the replication impairment imposed by the Y143C change. The reductions in infectivity that were observed for Y143C, E92Q+Y143C, T97A+Y143C, Q148H/K/R, and N155H ROD9 were not attributable to differences in transfection efficiency, since comparable titers were obtained from two or more independent preparations of plasmid DNA for each wild-type or mutant construct (Figure 1).

**Figure 2:**
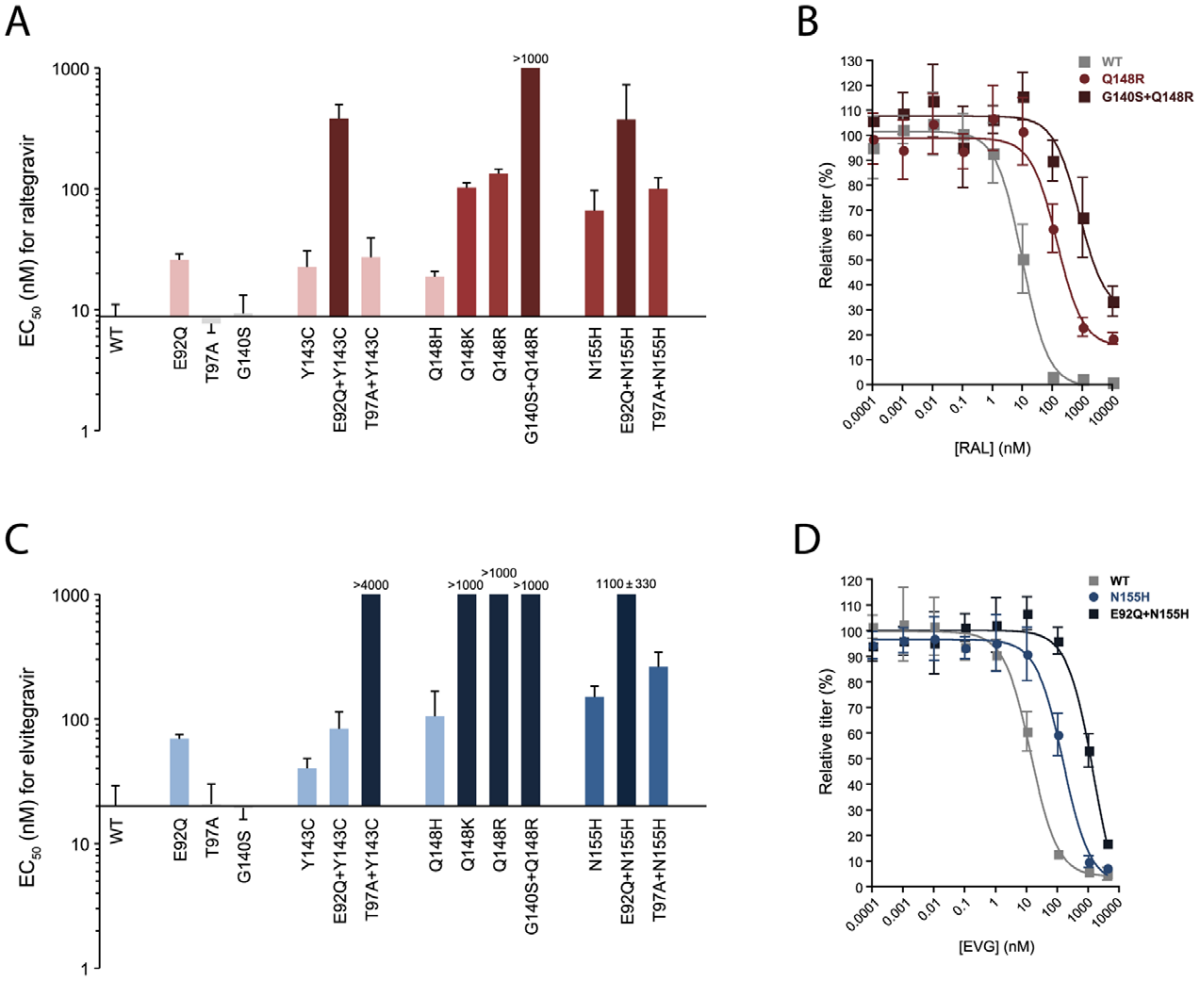
Susceptibility of HIV-2 integrase variants to raltegravir (RAL) and elvitegravir (EVG). Panels A and C show the EC_50_ values for wild-type (WT) HIV-2 ROD9 and each of 13 site-directed ROD9 integrase mutants tested against raltegravir and elvitegravir, respectively. Bars indicate the means of three or more independent dose-response experiments. Light, medium and dark-colored bars indicate low-level, moderate, and highlevel resistance (mean EC_50_ values 2–5-fold, 6–15-fold and >15-fold relative to wild-type, respectively). With the exception of Q148H versus raltegravir and Y143C versus elvitegravir, EC_50_ values for all strains shown in color were statistically greater than the corresponding values for wild-type ROD9 (*P*>0.05, ANOVA of log_10_-transformed EC_50_ values with Tukey's post-test). EC_50_ values for T97A and G140S did not statistically differ from WT for either drug. Panels B and D show representative dose-response data for WT, Q148R, and G140S+Q148R versus raltegravir and WT, N155H, and E92Q+N155H versus elvitegravir, respectively. Titers are expressed as the percentage of those seen in the absence of drug (i.e., % of solvent-only controls) and are the means of three independent cultures at each drug concentration. Error bars in all panels indicate standard deviations.

To evaluate the effects of each of the aforementioned amino acid changes on raltegravir susceptibility, wild-type and mutant virus stocks were used to infect MAGIC-5A indicator cell cultures that were treated with increasing concentrations of the inhibitor. Infected monolayers were then stained with X-gal to quantify the dose-dependent reduction of β-galactosidase–positive foci. Dose-response profiles for variants T97A, G140S and Q148H were similar to those obtained in parallel with wild-type ROD9 (Figure S1), resulting in mean EC_50_ values that were not significantly different from the parental strain (Figure 2A). Mutants E92Q, Y143C and T97A+Y143C showed statistically significant declines in raltegravir sensitivity, with mean EC_50_ values 3–4-fold greater than that of wild-type ROD9 (Figures 2A and S1). More substantial changes were conferred by Q148K, Q148R, N155H and T97A+N155H, which yielded moderate levels of resistance to the drug (7–15-fold; Figure 2A).

We also observed instances in which combinations of amino acid changes produced greater-than-additive increases in the level of raltegravir resistance. The G140S change alone produced no change in raltegravir susceptibility (Figure 2A), but the addition of G140S to the Q148R clone increased the level of raltegravir resistance from 15-fold (for Q148R alone) to >100-fold (for G140S+Q148R) (Figure 2B). Similarly, variants E92Q, Y143C and N155H were 3- to 7-fold resistant to the drug, but the double mutants E92Q+Y143C and E92Q+N155H exhibited mean EC_50_ values 43- and 42-fold greater than wild-type ROD9, respectively (Figure 2A). Taken together, these data show that replacements E92Q+Y143C, G140S+Q148R, and E92Q+N155H lead to high-level raltegravir resistance in HIV-2.

Lastly, we tested each of the variants in our panel for resistance to elvitegravir. Substitutions T97A and G140S had no effect on elvitegravir susceptibility, whereas replacements E92Q, E92Q+Y143C, Q148H, N155H, and T97A+N155H imparted low to intermediate levels of resistance to the drug (mean EC_50_ values 3–13-fold greater than wild-type; Figure 2C). Although not statistically significant, EC_50_ values for the Y143C variant were 2-fold greater than wild-type in each of three independent experiments, potentially indicating low-level elvitegravir resistance. Importantly, two combinations of amino acid changes yielded greater-than-additive increases in the level of resistance to elvitegravir: T97A+Y143C (>200-fold; Figure 2C) and E92Q+N155H (51-fold; Figure 2C and 2D). In contrast, replacements Q148K and Q148R alone were sufficient for a >50-fold increase in the EC_50_ for elvitegravir (Figure 2C). The combination of G140S+Q148R produced dose-response profiles similar to those of Q148R, although titers of the double mutant (expressed as % of no-drug controls) were consistently higher at 1000 nM elvitegravir, possibly reflecting a greater degree of resistance to the drug (Figure S1). Collectively, these data show that raltegravir-resistant mutants of HIV-2 are cross-resistant to elvitegravir and that substitutions T97A+143C, Q148K/R, G140S+Q148R and E92Q+N155H confer high-level elvitegravir resistance in HIV-2.

## Discussion

Our findings identify three main mutational pathways to high-level raltegravir and elvitegravir resistance in HIV-2. The first is centered around Y143C, which alone has only a modest effect on drug susceptibility. In the case of raltegravir, high-level resistance requires the combination of E92Q+Y143C, whereas T97A+Y143C is required for robust elvitegravir resistance. The second pathway is defined by G140S+Q148R, which yields high-level resistance to both drugs. While Q148R alone is sufficient for a dramatic loss of elvitegravir sensitivity, our data show that G140S cooperates with Q148R to restore replication capacity, and will therefore likely enhance viral fitness and escape in both raltegravir- and elvitegravir-treated HIV-2 patients. Finally, the combination of E92Q+N155H produces robust resistance to both raltegravir and elvitegravir in HIV-2, providing a third pathway to high-level resistance. Taken together, these findings preclude the sequential use of raltegravir and elvitegravir, or vice versa, for HIV-2 treatment and provide important information for clinical monitoring of INSTI resistance in HIV-2–infected individuals.

Further evidence for the roles of the aforementioned changes in INSTI resistance can be found in a recent study of the biochemical properties of wild-type and mutant HIV-2 integrase proteins. Specifically, Ni and colleagues [20] used a cell-free strand transfer assay to demonstrate that substitutions G140S+Q148R and N155H in HIV-2 integrase confer moderate to high-level resistance to raltegravir. In contrast, the Y143C change had little or no effect on raltegravir susceptibility; robust resistance to the drug required the addition of E92Q together with Y143C. Thus, while E92Q and G140S alone had no significant impact on raltegravir sensitivity, these replacements cooperated with Y143C and Q148R, respectively, to impart high-level raltegravir resistance in the strand transfer assay. Our results are concordant with these findings and additionally identify specific mutational pathways by which HIV-2 can acquire resistance to elvitegravir. The impact of resistance-associated mutations on the susceptibility of the HIV-2 to newer drugs in the INSTI class, such as dolutegravir, remains to be determined.

Although phenotypes similar to the ones described above have been reported for HIV-1 [19], it should be noted that the HIV-1 and HIV-2 integrase proteins differ at approximately one third of the 109 amino acid sites in the conserved catalytic domain, including sites implicated in INSTI resistance in HIV-1 [21], [22], [23] (alignments highlighting these differences are found in references 21 and 22). Thus, the phenotypes produced by amino acid changes in HIV-2 integrase cannot be predicted *a priori* based on their effects in HIV-1. In addition, the clinical implications of INSTI resistance in HIV-2 are potentially more profound compared to HIV-1, since other ARV classes are either ineffective against HIV-2 or provide minimal durability of treatment [4], [24] (although recent data suggest greater clinical success with regimens containing ritonavir-boosted proteases inhibitors [24], [25], [26]). Future efforts to improve treatment outcomes for HIV-2–infected patients should include evaluations of raltegravir- and elvitegravir-containing regimens in both ARV-naive and -experienced individuals, preferably in the setting of randomized clinical trials.

## Supporting Information

Figure S1 **Representative drug susceptibility data for raltegravir (RAL) and elvitegravir (EVG).** Each panel shows the results of a single experiment. Dose response profiles for wild-type HIV-2 ROD9 (filled squares) and each of the indicated HIV-2 integrase mutants (open squares) were determined in parallel. Titers are expressed as the percentage of those seen in the absence of drug (i.e., % of solvent-only controls) and are the means of three independent cultures at each drug concentration. Error bars are standard deviations.
(EPS)
